# CiteSpace-based visualization and analysis of hotspots and development trends in childbirth experience research

**DOI:** 10.3389/fgwh.2025.1590412

**Published:** 2025-11-06

**Authors:** Jie Shi, Yan Wang, Jieling Luo, Hailong Jiang, Xiaoting Geng, Mengyuan Xiang, Shuying Li

**Affiliations:** 1 School of Nursing, Chengde Medical College, Chengde, China; 2 Nursing Department, Affiliated Hospital of Chengde Medical College, Chengde, China

**Keywords:** maternity, birth experience, CiteSpace, knowledge graph, visual analytics

## Abstract

**Objective:**

To explore and analyze the current research status, hotspots, and development trend of labor and delivery experience, to provide a reference for subsequent related research and clinical practice.

**Methods:**

We searched the Web of Science database for literature related to labor and delivery experience published between its establishment and December 20, 2024, and conducted bibliometric analysis using CiteSpace software.

**Results:**

After screening, 1089 papers were included in the analysis, and the number of annual publications showed a growing trend, reaching its highest in 2024. The United States and Sweden dominated the list. The research hotspots focused on maternal mental health, delivery methods, and quality of Intrapartum care.

**Conclusion:**

The field of birth experiences is currently undergoing rapid development, with leading trends including innovations in delivery methods, prenatal care, research in the cognitive neuroscience of childbirth, and a focus on mothers undergoing induced labor and those in low-income areas to optimize the overall birth experience.

## 1 Introduction

Childbirth experience, defined as the subjective physical and psychological perceptions of women during parturition, represents a unique life event shaped by complex psychological and physiological processes influenced by societal, environmental, organizational, and policy factors (1). Empirical evidence reveals that 20%–48% of women experienced traumatic childbirth, with the majority reporting suboptimal experiences (2, 3). These negative experiences can trigger severe consequences, including postpartum depression, post-traumatic stress disorder (PTSD), and suicidality, while also damaging marital relationships, mother-infant bonding, and trust in healthcare providers. Furthermore, they contribute to tokophobia and pregnancy refusal, exacerbating global fertility decline (4–6).

In response, the United Nations Global Strategy for Women's, Children's and Adolescents' Health (2016–2030) and the World Health Organization Guidelines for the Management of Labor and Delivery (2018) have emphasized enhancing positive childbirth experiences as a primary goal of maternal healthcare services (7, 8). This has driven a significant increase in research over the past five years, which explored the status (9), influencing factors (10), and intervention strategies (11, 12) of childbirth experiences. However, the field of maternal childbirth experiences currently lacks a comprehensive framework that integrates research findings and identifies emerging trends.

To address this gap, we employed CiteSpace, a scientific literature analysis tool that visualizes research trends and highlights pivotal studies (13). Transforming complex data into visual knowledge maps offers researchers an innovative approach to understanding medical research advancements (14). In this study, we utilized CiteSpace to analyze maternal childbirth experience literature, identifying research hotspots, developmental trends, and field-specific patterns, thereby providing a foundation for future research.

## 2 Methods

### 2.1 Data collection and search strategy

We conducted a systematic literature retrieval from the Web of Science Core Collection (WoSCC), the premier multidisciplinary citation database containing over 21,000 high-impact journals across 254 disciplines. The search strategy employed Boolean operators: TS = (“birth experience” OR “childbirth experience” OR “labour experience” OR “delivery experience” OR “experience of childbirth” OR “birth satisfaction” OR “childbirth satisfaction” OR “delivery satisfaction”).

**Figure 1:**
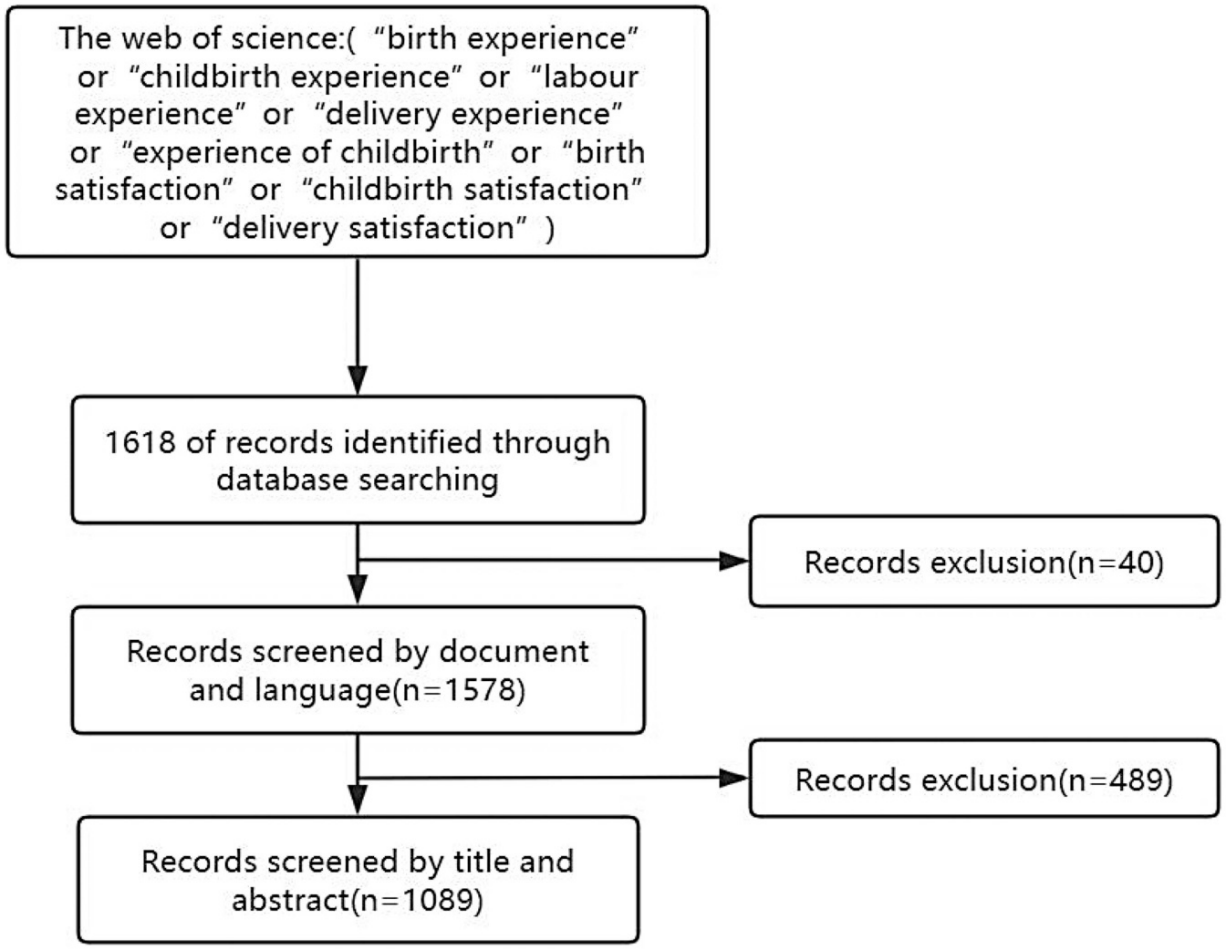
Flow chart of literature selection.

The search parameters were constrained by: Temporal scope: Inception to December 20, 2024 (executed December 20, 2024, to eliminate daily update bias), Document types: Articles and reviews, and Language: English. This strategy yielded 1,089 eligible records after removing duplicates and non-conforming publications (Figure 1). Full bibliometric data including cited references were exported in plain text format for CiteSpace compatibility.

### 2.2 Analytical framework

CiteSpace 6.1.R2 was used for the visual analysis. Export the imported documents in “.txt” format, then save them in download_txt format to the input folder, and then automatically convert the format to download_converted.txt and save it to the output folder. Import the data into CiteSpace and set the relevant parameters: the time span is from 1975 to 2024 and the time slice is set to 1 year. The selection criterion was set as TOPN, the threshold value was 50, and the clipping method was selected as the Pathfinder method. A knowledge map of the research on maternal birth experience was created.

## 3 Results

### 3.1 Trends in the annual number of publications

**Figure 2:**
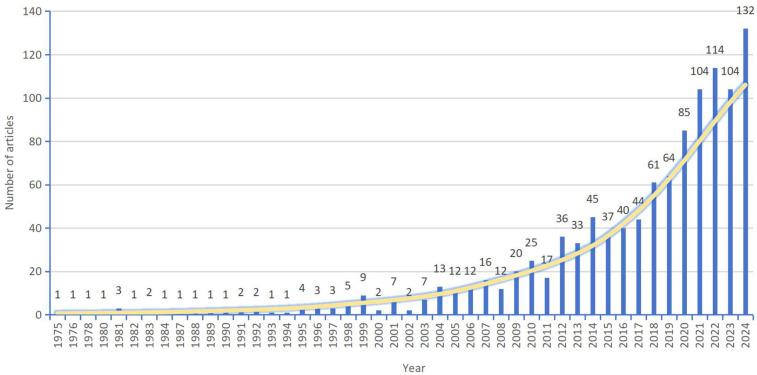
Trends in annual publications in the literature related to the childbirth experience.

The trend of annual publications in the field of maternal birth experience from 1975 to 2024 is shown in Figure 2, which shows that the first publication in the field of birth experience appeared in 1975, and the number of publications from 1975 to 2003 was relatively small, with fewer than 10 publications; the number of publications from 2003 to 2017 gradually increased; the number of publications from 2017 onwards increased rapidly and reached a peak of 132 publications in 2024. This sustained and accelerating growth, particularly the sharp rise in recent years, indicates that maternal birth experience is evolving into a dynamic and increasingly prominent research area, likely to attract continued scholarly attention and further investigation in the foreseeable future.

### 3.2 Distribution of literature published by countries and organizations and analysis of collaborative networks

**Figure 3:**
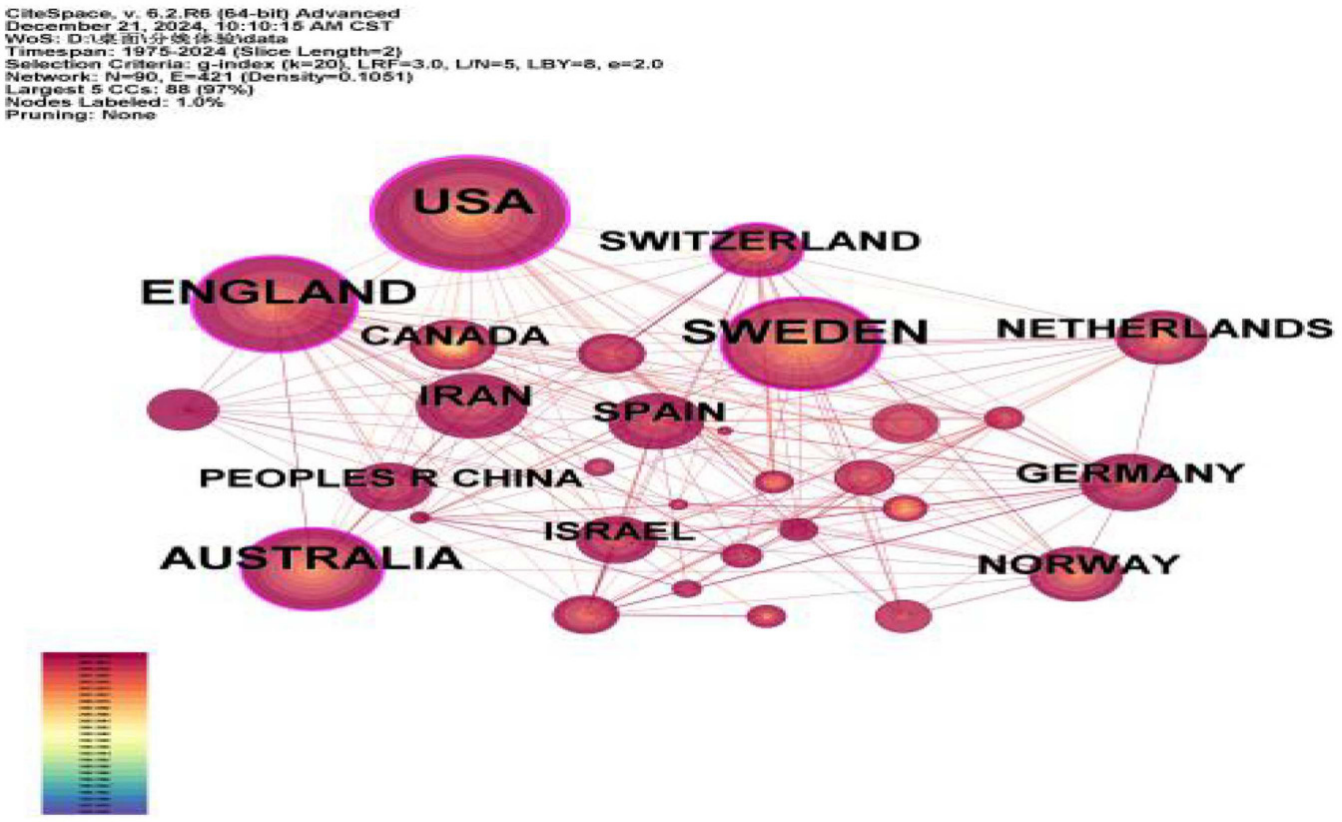
Mapping of national cooperation in the literature.

According to the analysis of Table 1 and Figure 3, it can be seen that a total of 90 countries have published literature related to childbirth experience, of which the United States, Sweden, the United Kingdom, and Australia are the core output forces, accounting for 56.11% of the total output, and the top 10 countries with the highest number of issuance account for 84.31% of the total number of issuance; the United States and Sweden are more centrally oriented and have a greater influence. In the co-authorship network, the United States and Sweden exhibit high centrality, indicating their roles as major hubs for international collaboration and their significant influence within the global research landscape of maternal childbirth experience.

**Table 1:** Top 10 countries and institutions in terms of annual publications in the literature related to the childbirth experience.

Ranking	Country	Count	Centrality	Institution	Count	Centrality
1	USA	202	0.57	Karolinska Institutet	71	0.22
2	SWEDEN	144	0.39	Tabriz University of Medical Science	34	0.13
3	ENGLAND	44	0.16	Uppsala University	32	0.04
4	AUSTRALIA	118	0.15	University of Liverpool	24	0.03
5	IRAN	151	0.1	University of London	23	0.27
6	GERMANY	45	0.09	University of Gothenburg	22	0.11
7	NORWAY	50	0.07	Mid-Sweden University	21	0
8	NETHERLANDS	47	0.07	Griffith University	18	0.02
9	SPAIN	38	0.07	University of Central Lancashire	16	0.05
10	CANADA	22	0.07	Norwegian Institute of Public Health (NIPH)	15	0.04

**Figure 4:**
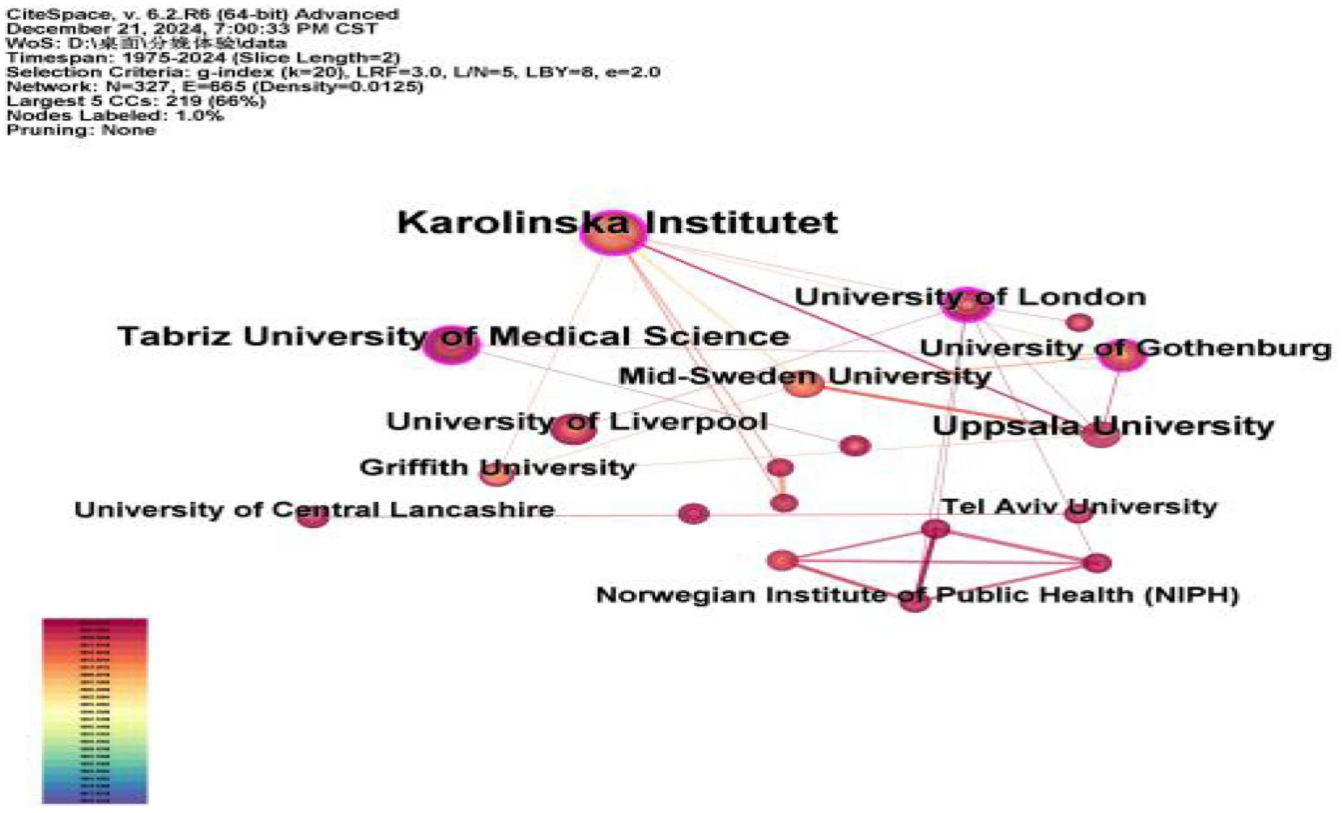
Institutional collaborative Map of literature.

According to the analysis of Table 1 and Figure 4, it can be seen that a total of 327 institutions have published literature related to childbirth experience, and the institution with the most publications is Karolinska Institutet in Sweden, which has published 71 articles, and the institution with the strongest centrality is the University of London in the UK, which has published 23 articles and has the most prominent academic influence. As far as the inter-institutional cooperation relationship is concerned, there are 665 connections of 327 nodes, with a network density of 0.0125, indicating that the cooperation and exchange between institutions is not sufficient and needs to be deepened.

### 3.3 Analysis of cited journals

**Figure 5:**
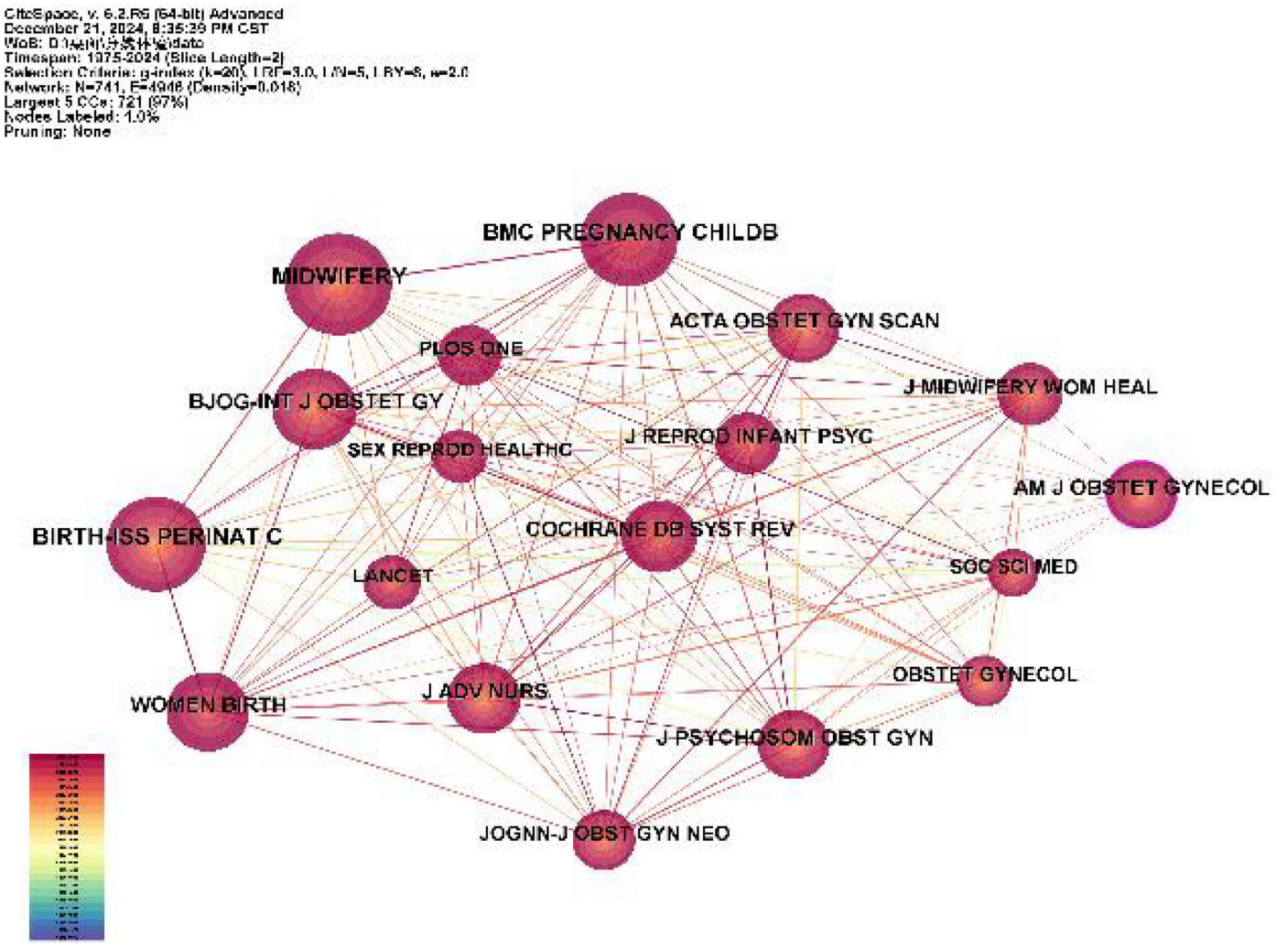
Collaborative mapping of the top 10 cited journals.

According to the analysis in Table 2 and Figure 5, there are 741 journals published in the literature related to childbirth experience, most of which are in Europe and America. The most cited journals are *MIDWIFERY* (impact factor of 2.6) with 785 citations, followed by *BIRTH-ISS PERINAT C* (impact factor of 2.8) with 759 citations. Among them, *AM J OBSTET GYNECOL* (with an impact factor of 8.7) has the strongest centrality and the greatest academic impact. As far as the collaboration between journals is concerned, there are 4,946 links of 741 nodes with a node density of 0.018, which indicates that there is less collaboration between journals.

**Table 2:** Top 10 journals cited in literature related to the childbirth experience.

Ranking	Journal	Impact Factor	Count	Centrality
1	MIDWIFERY	2.6	785	0.04
2	BIRTH-ISS PERINAT C	2.8	759	0.07
3	BMC PREGNANCY CHILDB	2.8	629	0.02
4	BJOG-INT J OBSTET GY	4.7	500	0.01
5	WOMEN BIRTH	4.4	453	0.01
6	ACTA OBSTET GYN SCAN	3.5	426	0.02
7	J PSYCHOSOM OBST GYN	2.1	412	0.02
8	AM J OBSTET GYNECOL	8.7	409	0.11
9	COCHRANE DB SYST REV	8.8	393	0.02
10	J ADV NURS	3.8	389	0.02

### 3.4 Analysis of cited literature

From the analysis of Table 3, it can be seen that the most cited literature is “The birth experience and women's postnatal depression: A systematic review” (93 citations), which systematically evaluates the relationship between labor and delivery experience and postnatal depression, and suggests that poor labor and delivery experience can cause maternal postnatal depression. This was followed by “The etiology of post-traumatic stress following childbirth: a meta-analysis and theoretical framework” (cited 81 times) and “The prevalence of posttraumatic stress disorder in pregnancy and after birth: a systematic review and meta-analysis” (cited 58 times), the above two papers analyzed the relationship between post-traumatic stress disorder and childbirth experience, and the analysis concluded that adverse childbirth experience was the greatest predictor of postpartum PTSD.

**Table 3:** Top 10 cited documents.

Ranking	Title	Author	year	Count	Journal
1	The birth experience and women's postnatal depression: A systematic review	Bell AF	2016	93	MIDWIFERY
2	The etiology of post-traumatic stress following childbirth: a meta-analysis and theoretical framework	Ayers S	2016	81	Ayers S
3	The prevalence of posttraumatic stress disorder in pregnancy and after birth: A systematic review and meta-analysis	Yildiz PD	2017	58	J AFFECT DISORDERS
4	The meaning of a very positive birth experience: focus groups discussions with women	Karlström A	2015	57	BMC PREGNANCY CHILDB
5	The Mistreatment of Women during Childbirth in Health Facilities Globally: A Mixed-Methods Systematic Review	Bohren MA	2015	52	PLOS MED
6	Factors related to a negative birth experience—A mixed methods study	Henriksen L,	2017	49	MIDWIFERY
7	Prevalence and risk factors of postpartum posttraumatic stress disorder: a meta-analysis	Grekin R	2014	48	CLIN PSYCHOL REV
8	Predictors of a negative labour and birth experience based on a national survey of Canadian women	Smarandache A	2016	48	BMC PREGNANCY CHILDB
9	Continuous support for women during childbirth	Hodnett ED,	2011	47	COCHRANE DB SYST REV
10	Measuring women's childbirth experiences: a systematic review for identification and analysis of validated instruments	Nilvér H	2017	44	Helena Nilvér

### 3.5 Analysis of author publication volume

**Figure 6:**
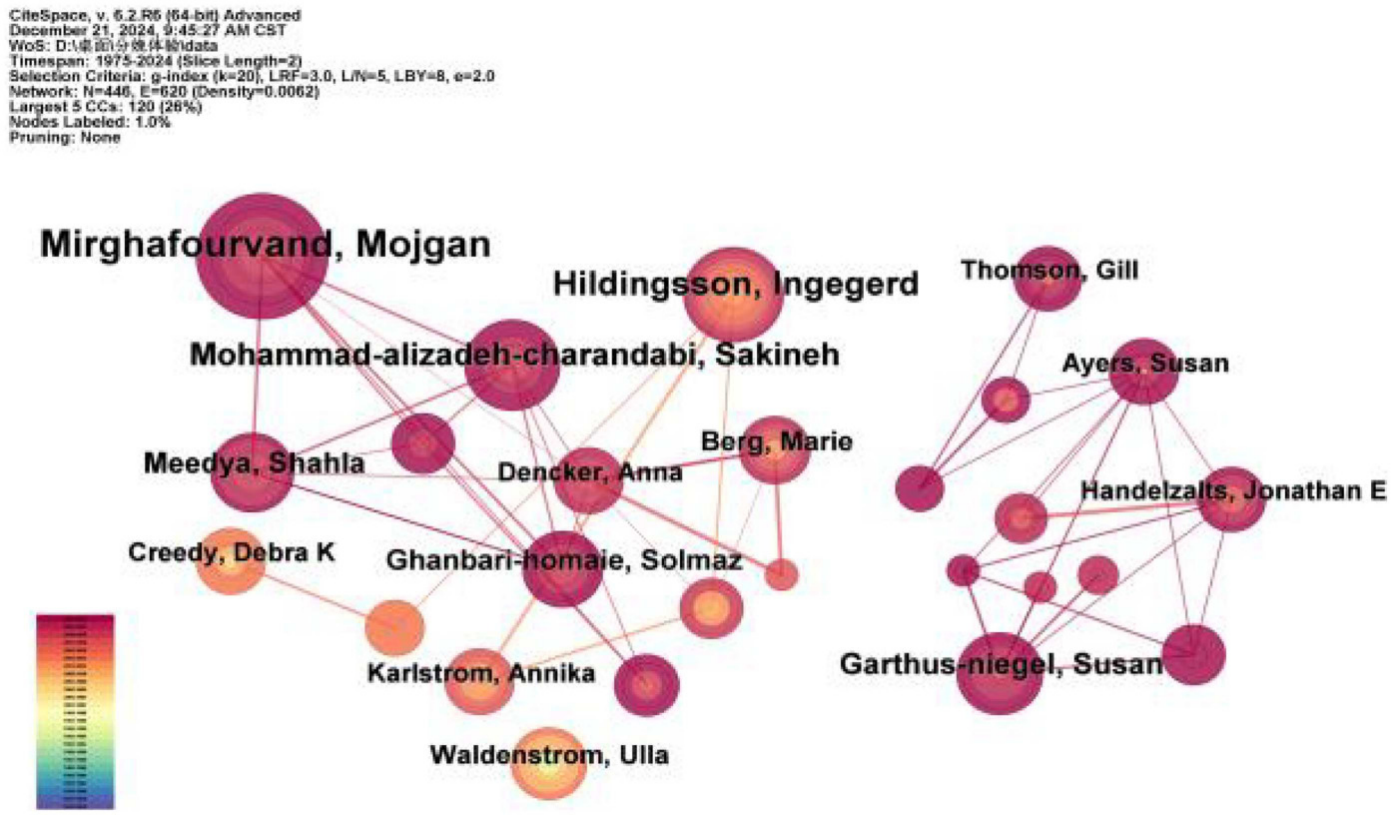
Collaborative network mapping of top 10 authors in terms of publications.

As shown in Table 4 and Figure 6, a total of 446 authors have published literature in the field of childbirth experience. The most prolific author is Mojgan Mirghafourvand, from Ireland, with 31 publications. The co-authorship network, comprising 446 nodes and 620 links, has a density of 0.0062, indicating a generally sparse collaborative structure. Analysis reveals a primary collaborative cluster centered around Mojgan Mirghafourvand, who maintains strong ties with co-authors such as Sakineh Mohammad-alizadeh-Charandabi and Shahla Meedya. A secondary cluster is formed around Solmaz Ghanbari-Homayi and other core authors. However, collaborative activities beyond these core groups remain limited. The future promotion of wider-ranging collaboration is crucial for integrating diverse expertise and tackling the complex, multifaceted nature of research on childbirth experiences.

**Table 4:** Top 10 authors in terms of publications.

Ranking	Author	Count
1	Mirghafourvand, Mojgan	31
2	Mohammad-alizadeh-charandabi, Sakineh	17
3	Garthus-niegel, Susan	14
4	Meedya, Shahla	13
5	Ghanbari-homaie, Solmaz	12
6	Berg, Marie	11
7	Waldenstrom, Ulla	10
8	Creedy, Debra K	9
9	Karlstrom, Annika	9
10	Ayers, Susan	9

### 3.6 Keyword analysis

#### 3.6.1 Keyword Co-occurrence analysis

Keywords are a high degree of summary and condensation of the theme of the literature, and by analyzing the high-frequency keywords of the literature, we can reveal the hotspots and tendencies of the research in this field and the relationship between the research themes. After combining the synonyms, the keywords are ranked in the keyword map in Table 5, and the words with the highest frequency are “childbirth”, “birth experience”, 651 and 576 times respectively, followed by the words “risk factors”, “depression”, “posttraumatic stress”, and so on. “risk factors”, “depression”, “posttraumatic stress disorder”; the words with centrality ≥0.1 were “birth satisfaction”, “child birth”, and “pregnancy”, indicating that they are hot topics in the study of birth experience.

**Table 5:** Top 10 keywords in terms of frequency and centrality of occurrence.

Ranking	Frequency	Keyword	Ranking	Centrality	Keyword
1	651	childbirth	1	0.11	birth satisfaction
2	576	birth experience	2	0.1	childbirth
3	196	risk factors	3	0.1	pregnancy
4	195	depression	4	0.09	depression
5	181	posttraumatic stress disorder	5	0.09	perceptions
6	181	women	6	0.09	mothers
7	173	care	7	0.08	cesarean section
8	170	pregnancy	8	0.08	outcom
9	169	prevalence	9	0.07	care
10	164	cesarean section	10	0.07	anxiety

#### 3.6.2 Keyword cluster analysis

**Figure 7:**
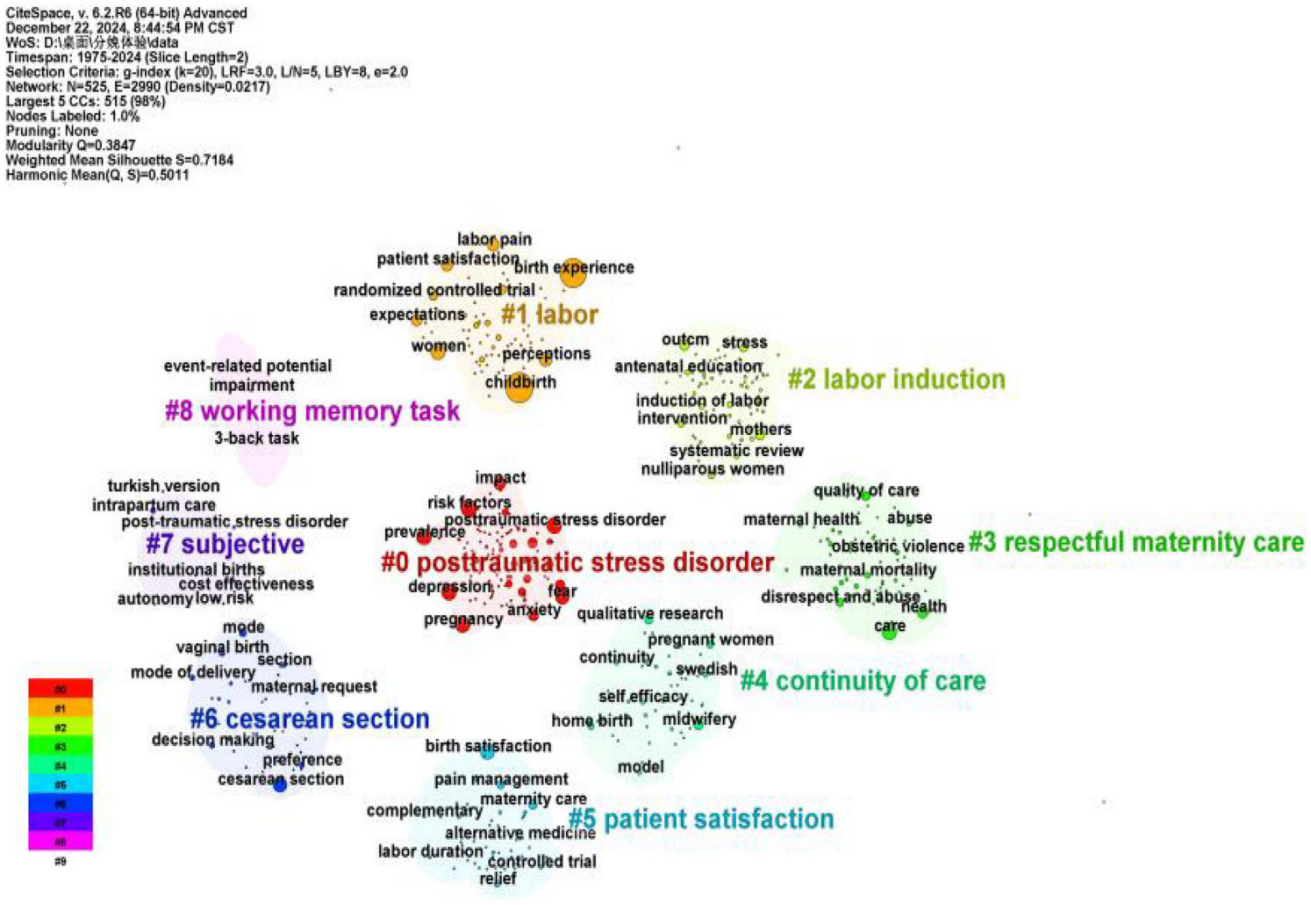
Cluster analysis diagram.

Keyword clustering is a method of grouping multiple similar keywords into a single label using induction, which reflects the research hotspots in the research process and helps to identify future research directions in the field. According to the analysis in Table 6 and Figure 7, a total of nine clusters were generated for keywords related to childbirth experience, and the clustering results showed that the Q value was 0.3847 and the S value was 0.7184, which made the clusters plausible. The clustering keywords were mainly “posttraumatic stress disorder” “labour” “labour induction” “respectful maternity care” “continuity of care” “patient satisfaction” “cesarean section” “subjective” and “working memory task”. The areas covered include factors influencing the maternal birth experience, assessment tools, quality of care, and research methods.

**Table 6:** Cluster analysis.

Cluster number and name	Number of documents	Silhouette value	High impact words
#0posttraumatic stress disorder	102	0.726	fear of childbirth; ptsd; revalence; symptoms
# 1 labor	82	0.684	expectations; satisfaction; pain; epidural analgesia
# 2 labor induction	82	0.654	induction of labor; birth environment; systematic review; psychometric properties
# 3 respectful maternity care	69	0.712	maternal health; disrespect; abuse; quality of care
# 4 continuity of care	54	0.648	qualitative research; home birth; patient safety; childbirth humanization
# 5 patient satisfaction	54	0.808	validation studies; birth satisfaction; scales; shift work
# 6 cesarean section	46	0.769	vaginal birth; cesarean delivery; maternal request; mode of delivery
# 7 subjective	10	0.916	intrapartum care; validated questionnaires; utility; positive childbirth
# 8 working memory task	8	0.998	2-back task; event-related potential; 1-back task; childbirth

#### 3.6.3 Keyword emergence analysis

**Figure 8:**
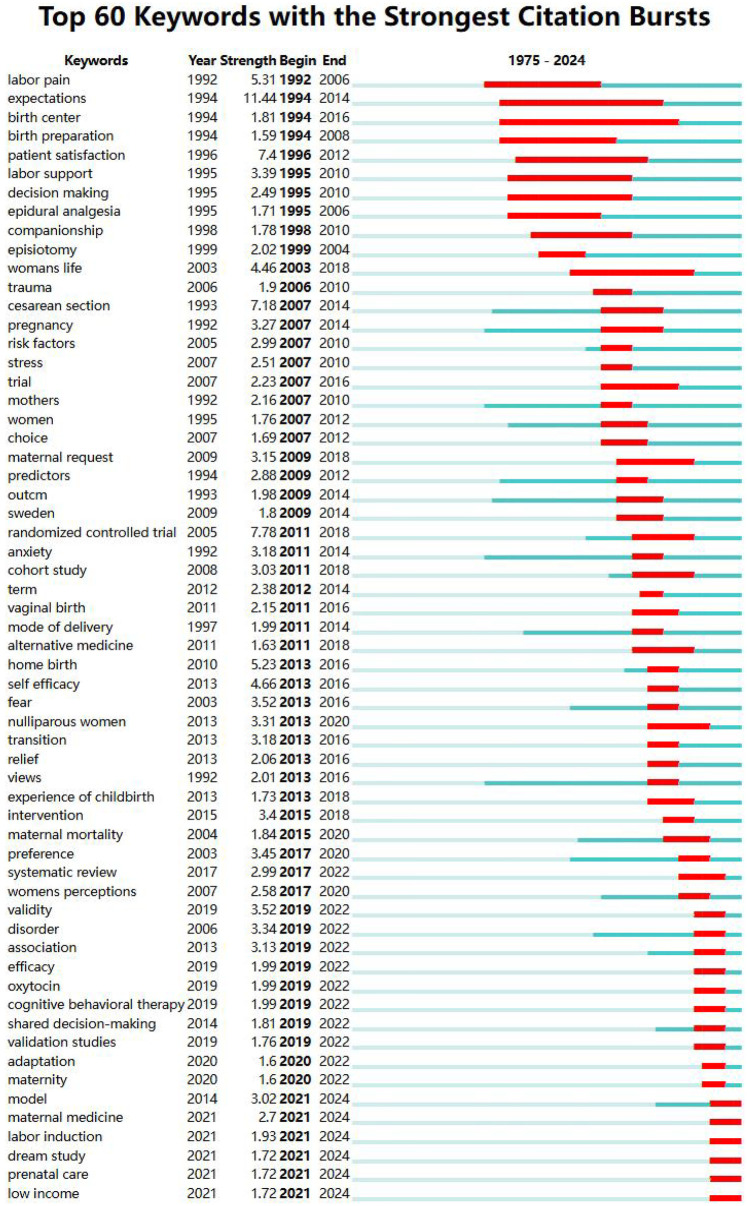
Analysis of emergent words.

The emergent words are the keywords with high frequency in a period, showing the intensity, rise, or decline of a keyword emergence, which can describe the evolution process and development trend of the research frontiers and predict the future research direction. The burstness function generates a map of emergent words, and the high-frequency keyword emergence analysis (*γ* = 0.4) maps out 60 emergent words appearing from 1975 to 2024, see Figure 8. In this figure, keywords are arranged in a vertical timeline, with earlier years at the top and more recent years at the bottom, illustrating the evolution of research fronts over time. The longest time span is the study of birth centre, and the related research lasted from 1994 to 2016. The highest intensity of emergence was for “expectations” with the highest emergence (11.44), followed by “randomised controlled trial,” “patient satisfaction” and “cesarean”. It is worth noting that “model”, “maternal medicine”, “labour induction”, “dream study”, “prenatal care”, and “low income” are key words that appeared only in recent years, which may become the hotspot and trend of research in the future and will attract more researchers to pay extensive attention to them.

## 4 Discussion

### 4.1 Current status of the study

Analyzed by the yearly trend graph of the number of publications, this research field was in the beginning stage from 1975 to 2003, in the steady development stage from 2003 to 2017, and in the rapid development stage from 2017. Possible reasons for this are: (1) the development of medical technology: new technologies such as virtual reality (VR) have provided new ways and methods to improve the childbirth experience (15–17); (2) the advancement of global health policies, and in 2016, WHO released the Framework for Improving Maternity Care, which for the first time included the 'satisfaction of the maternal experience' as a core indicator of healthcare quality (18); (3) increased social attention to women's health and reproductive rights, as well as increased maternal expectations of the birth experience (19).

From the analysis of the graphs of countries, institutions, journals, and authors of published literature, it can be seen that developed countries such as Europe and the United States have a high volume of publications, high influence, and close cooperation, while Asian, African, and South American countries pay less attention to this field and have less cooperation, and this unbalanced mode of international cooperation may be related to the advancement of languages, cultures, medical technologies, medical security systems, and the importance of maternal health. The more economically developed a country is, the more enthusiastic it is about research on the maternal birth experience. It is recommended that countries, institutions, and authors strengthen international cooperation and balance the allocation of resources to promote research.

### 4.2 Research hot spots

Combining the high-frequency keyword list, keyword co-occurrence mapping, keyword clustering chart, emergent keyword list, and high-frequency cited literature list, and also combining the specific content of the literature, this paper believes that the hot topics of research on childbirth experience can be categorized into the following three aspects.

#### 4.2.1 Mental health and childbirth experience: an in-depth analysis

The high-frequency keywords, clusters, and emergent words “fear,” “depression,” and “posttraumatic stress disorder” indicate that mental health is one of the research hotspots in the field of labour experience. The special process of labour involves not only huge changes on the physical level but also poses many challenges on the psychological level. Lack of knowledge about childbirth, severe pain during labour, unexpected situations, and even the words and attitudes of healthcare professionals may all be triggering factors for posttraumatic stress disorder and fear of childbirth (20–23), which significantly increase the probability of posttraumatic stress disorder, fear of childbirth, and postnatal depression, and these negative experiences are like shadows, which continue to affect the mother's physical and mental health and family relationships. Currently, maternal mental health has gradually attracted the attention of scholars in various countries, and the UK government has also established the Childbirth Trauma Association and set up a national cooperation with professionals to “make childbirth better”, which manages all mothers and their partners who report traumatic birth experiences (24). The nursing staff is advised to assess the psychological state of the mother before and during labour and to encourage her to share her experience of childbirth, to use various psychological support methods to channel negative emotions, and, if necessary, to make timely referrals to psychosocial services for mothers at risk of psychiatric disorders.

#### 4.2.2 Different modes of delivery and birth experience: a comparative analysis

The high-frequency keywords such as “cesarean section,” “vaginal birth,” and “induced labor” underscore researchers' focus on the relationship between delivery modes and labor experience. A 2013 study (25) highlighted that the mode of delivery itself does not directly influence maternal satisfaction with labor. Instead, key factors such as participation in decision-making, support during labor, and effective analgesia play a pivotal role in enhancing the labor experience. However, recent studies (26, 27) have demonstrated that different delivery modes significantly impact maternal postpartum satisfaction, with vaginal deliveries generally yielding higher satisfaction levels compared to cesarean deliveries.

Future research should focus on conducting multi-center, large-sample, systematic studies to explore in depth the association between different modes of delivery and maternal birth experience. At the same time, individualized intervention strategies should be developed to address the characteristics of different modes of delivery, to effectively improve maternal satisfaction and the overall delivery experience.

#### 4.2.3 Enhancing intrapartum care quality

The high-frequency keywords such as “respectful maternity care,” “continuity of care,” and “patient satisfaction” underscore the importance of improving care quality as a key research priority in the field of childbirth experience. A systematic evaluation by Bohren (28) revealed that mistreatment of women during labor and delivery is widespread in global health facilities, encompassing physical and verbal abuse, stigmatization, and non-consensual medical interventions. Such mistreatment significantly impacts maternal labor and delivery experiences negatively.

To address this issue, national guidelines emphasize the importance of respecting the birthing population and providing continuous support throughout labor. For example, the World Health Organization (WHO) guideline “Managing Birth to Improve the Birth Experience” (2018) (8) emphasizes the importance of preserving the dignity, privacy, and confidentiality of all women in labor. It asserts that women should be protected from harm and wrongful interventions, have access to informed choices, and receive ongoing support throughout labor and delivery. Similarly, the 2023 NICE guidelines on care during labor (29) stress the need for continuous support and advocate for one-to-one support during childbirth. Studies (30, 31) have shown that respectful maternity care can alleviate maternal fear and anxiety about childbirth and promote a positive birth experience by reducing the incidence of pain medication, vaginal assisted delivery, cesarean section, and dissatisfaction with the birth experience. It is recommended that countries promote and implement respect and continuum of care support by with the guidelines, strengthen healthcare training, and engage families to provide holistic care for women in labour.

### 4.3 Research frontiers and trends

#### 4.3.1 Innovative delivery models

The keyword “model” in the emergence chart has emerged more frequently in recent years. This may be due to the increasing attention paid by society to women's health and reproductive rights, as well as the rising expectations of mothers for the birth experience, which have led to innovations in birth models. Examples include home-based birthing centers and personalized birthing plans, which emphasize the autonomy and comfort of the mother. Studies (32, 33) have shown that this model allows women to better express their needs and preferences, reduces unnecessary medical interventions, lowers cesarean section rates, and improves the overall birth experience. However, research in this area still needs to be deepened, especially in terms of cultural adaptation, long-term effects, and policy translation. Future research should focus more on multidisciplinary collaboration and global perspectives to provide more comprehensive and personalized support for women's birth experiences.

#### 4.3.2 Antenatal care

In recent years, prenatal care has received much attention as one of the central themes in research on the experience of childbirth. High-quality prenatal care not only reduces the risk of complications during pregnancy but also significantly improves the labor experience and postpartum mental health (34). However, the traditional antenatal care model mainly focuses on the biomedical level, focusing on screening and diagnosis of diseases during pregnancy, and pays insufficient attention to the psychological and social support of pregnant women, which fails to adequately meet the diverse and individualized health needs of pregnant women, resulting in low participation and satisfaction of pregnant women in antenatal care (35, 36). In recent years, the centralized Group-Based Prenatal Care (GBPC) model has gradually received international attention, which emphasizes that pregnant women are the main body of health care and promotes active participation of pregnant women in health care using of group interactions and peer support (37), and can significantly improve pregnant women's satisfaction with pregnancy and delivery experience. In addition, with the rapid development of information technology, digital and intelligent antenatal care models have gradually become a research hotspot. Through mobile health applications, remote monitoring devices, and virtual reality (VR) technology, pregnant women can monitor their own and their fetus's health status in real-time at home and receive support from professional medical teams (38, 39). This model not only improves the accessibility and convenience of prenatal care but also significantly improves pregnant women's pregnancy experience through personalized health education and psychological support. However, as both are still in the developmental stage, there are still some limitations in related studies. Therefore, more rigorously designed studies with adequate sample sizes are needed to further validate their long-term effects and applicability.

#### 4.3.3 Cognitive neuroscience research

In recent years, cognitive neuroscience has increasingly contributed to the study of labor and delivery experiences. Researchers have employed advanced cognitive task techniques, including the “working memory task,” the “2-back task,” and “event-related potentials,” to investigate the psychological and physiological mechanisms underlying childbirth. These methodologies provide valuable insights into the neural basis of maternal cognition and emotional regulation during birth.

For instance, Olza (40) demonstrated that the release of endogenous oxytocin during labor induces neurobiological processes that influence maternal behavior and emotions, thereby facilitating the labor process. Additionally, their findings suggest that the psychological experiences during labor may play a crucial role in the successful transition to motherhood. Another study (41) highlighted that working memory performance during labor is susceptible to various factors, such as pain intensity, hormonal fluctuations, and psychological stress. Furthermore, event-related potentials have proven effective in capturing the precise temporal and neural correlates of maternal brain responses to specific stimuli, such as contraction perception and fetal status monitoring (42).

To advance this field, future research should prioritize interdisciplinary collaboration, integrating expertise from psychology, obstetrics, and neuroscience. Moreover, there is a need to translate neuroscience-based interventions into clinical practice through rigorous validation in large-scale trials.

#### 4.3.4 Focus on the maternal experience of childbirth in Low-income areas

In recent years, the keyword “low income” has emerged prominently in research on the experience of childbirth, suggesting that many scholars have begun to pay attention to the experience of childbirth in low-income areas. Global health equity requires that low-income maternal experience of childbirth be included as a core indicator in the Sustainable Development Goals. Existing evidence suggests that: maternal mortality rates in low-income areas are 22 times higher than in developed countries (43); less than 50 percent of deliveries in sub-Saharan Africa take place in healthcare facilities, and deliveries are significantly less safe than those in higher-income groups (44); and meta-analysis confirms that low-income mothers have a 2.4-fold increase in the risk of post-partum depression, which has a dose-response relationship with traumatic birth experiences (45).

In the future, it is recommended to make breakthroughs in the following directions: (1) technological innovation and sinking: the promotion of low-cost decision-supporting mobile health (such as AI midwife system), a pilot project in Nigeria proved that it can reduce maternal mortality (46); (2) cross-sectoral collaboration mechanisms: the establishment of a government-community-health-care institution tripartite linkage of the birth experience improvement program, the Kerala model in India, which has led to a significant increase in the rate of institutional births (47, 48); (3) Training of midwives at the grassroots level: studies have shown that for every additional midwife with standardized training, there is a reduction in maternal mortality (49).

#### 4.3.5 Focus on issues related to induced labor

In recent years, “labor induction” has emerged as a prominent keyword in childbirth experience research. As a pivotal intervention in modern obstetrics, labor induction is essential for managing specific maternal complications, such as gestational hypertension and gestational diabetes (50); however, its application faces two systemic challenges. First, clinical indications lack standardization, particularly in resource-limited settings. Inconsistent assessment often leads to poorly timed or unnecessary induction, increasing maternal discomfort and the risk of cascading interventions such as cesarean delivery (51). Second, beyond clinical standardization issues, the maternal experience itself is frequently overlooked. A focus on procedural adherence over woman-centered care creates communication gaps and psychological distress—both of which may adversely affect birth satisfaction and postpartum mental health. The absence of international quality standards further impedes consistent, high-quality care (52).

To address these issues, international bodies should develop evidence-based global guidelines, supported by digital training tools to improve implementation—especially in resource-limited settings where inconsistent assessment is most prevalent. Simultaneously, a woman-centered model must be adopted—ensuring transparent communication, shared decision-making, and empathetic support throughout the induction process.

#### 4.3.6 From childbirth event to maternal lifelong health

Emergence analysis reveals that “Maternal Medicine” has emerged as a leading focus in recent years within the field of childbirth experiences. While traditional research predominantly centered on the physiological and psychological events experienced by mothers during delivery, the advent of maternal medicine has broadened the research perspective beyond the singular event of childbirth (53). It now encompasses a comprehensive concern for the long-term health and quality of life of mothers as whole individuals (54). This shift signifies the field's evolution from a single discipline focused solely on ensuring delivery safety to a comprehensive discipline dedicated to optimizing health outcomes throughout the entire pregnancy period and beyond. Under this new paradigm, the concept of birth experience gains deeper significance: it is no longer defined solely by labor support, pain management effectiveness, or healthcare provider attitudes, but encompasses a continuum of care spanning preconception counseling, management of pregnancy complications, and long-term postpartum health follow-up.

Consequently, future research must prioritize establishing continuous care pathways throughout the perinatal period and integrate maternal long-term health indicators and quality of life into core evaluation systems.

### 4.4 Limitations of the study

This study has several limitations that should be acknowledged. First, the findings are constrained by the data sources and methodology. The analysis relied exclusively on English-language articles indexed in the Web of Science Core Collection. Therefore, it may not capture relevant studies published in other languages or housed in regional databases. Moreover, as a common limitation in bibliometric analyses, our dataset likely omits the very latest research that is not yet publicly available or indexed, such as preprints, ongoing clinical trial results, and gray literature. This may affect the immediacy of the identified frontiers. Second, potential geographic bias exists. Significant disparities in economic power and population size among countries can influence research output and focus, which may introduce bias in the global landscape portrayed here. Despite these limitations, this study provides a valuable baseline assessment.

## 5 Summary

In this study, CiteSpace software was used to conduct a comprehensive visual analysis of the literature related to childbirth experience in the Web of Science database. By analyzing the trend of the number of publications, the distribution of countries and institutions, keywords, and other aspects, the study reveals the current research status, hotspots, and development trends in this field. It is found that the number of publications in the field of labor and delivery experience continues to grow, with the United States and other Western countries as the main research force. The research hotspots are centered on maternal mental health, delivery mode, and quality of intrapartum care, and the research on innovation of delivery mode, antenatal care, cognitive neuroscience of delivery, and research focusing on the birth experiences of mothers in low-income areas and those undergoing induced labor will become future research frontiers and development trends. The results of this study provide a reference for future research in the field of labor and delivery experience, and at the same time, lay a foundation for further in-depth investigation of the theory and practice of labor and delivery experience, to meet the needs of mothers for a quality labor and delivery experience and to promote the health of mothers and infants. Future research needs to expand the sample globally, strengthen cross-cultural research, and emphasize the use of multidisciplinary theories and methods to deeply analyze issues related to the childbirth experience, to further promote the development of this field.
